# Supplementary material for: Characterization of GPX Gene Family in Pepper (Capsicum annuum L.) under Abiotic Stress and ABA Treatment
Source: Int J Mol Sci. 2024 Jul 30;25(15):8343. doi: 10.3390/ijms25158343 (PMC11313330; doi:10.3390/ijms25158343)
Supplement: Supplementary file 1 [file ijms-25-08343-s001.zip › Supplementary Materials.pdf]

Figure S1: The tertiary structure of CaGPX family members;

Figure S2: Phenotypes of pepper plants under different treatments;

Table S1: Secondary structure and subcellular localization prediction of the eight pepper CaGPX proteins;

Table S2: Ka/Ks values of *CaGPX* gene pairs in peppers and *Arabidopsis*;

Table S3: Conserved motifs in the amino acid sequences of CaGPX proteins;

Table S4: Identification of cis-regulatory elements in the pre 2000 bp region of the pepper *GPX* gene promoter;

Table S5: The GO enrichment analysis of *CaGPX* genes;

Table S6: CaGPXs protein interaction analysis;

Table S7: The protein sequence of *GPX* family genes in pepper, *Arabidopsis thaliana*, rice, cucumber, watermelon, apple, and *Rhodiola crenulata*;

Table S8: The sequences of primers;

Additional File S1: Amino acid substitution model for phylogenetic trees;

Additional File S2: Raw data of qRT-PCR.
