# Supplementary material for: Characterization of GPX Gene Family in Pepper (Capsicum annuum L.) under Abiotic Stress and ABA Treatment
Source: Int J Mol Sci. 2024 Jul 30;25(15):8343. doi: 10.3390/ijms25158343 (PMC11313330; doi:10.3390/ijms25158343)
Supplement: Supplementary file 1 [file ijms-25-08343-s001.zip › Table S1.pdf]

**Table S1.** Secondary structure and subcellular localization prediction of the 8 pepper CaGPX proteins

| Gene ID | Gene name | $\alpha$-helix % | Beta turn % | Random coil % | Extended strand % | Subcellular localization |
|---|---|---|---|---|---|---|
| CA.PGAv.1.6.scaffold774.11 | GPX1 | 28.99 | 11.24 | 37.28 | 22.49 | Chloroplast |
| CA.PGAv.1.6.scaffold273.17 | GPX2 | 19.2 | 6.4 | 44.8 | 29.6 | Nucleus |
| CA.PGAv.1.6.scaffold65.187 | GPX3 | 30 | 9 | 38.5 | 22.5 | Cytoplasm |
| CA.PGAv.1.6.scaffold1682.18 | GPX4 | 27.06 | 10.59 | 38.82 | 23.53 | Chloroplast |
| CA.PGAv.1.6.scaffold609.2 | GPX5 | 26.47 | 10.59 | 40 | 22.94 | Chloroplast |
| CA.PGAv.1.6.scaffold26.15 | GPX6 | 26.04 | 12.43 | 37.87 | 23.67 | Cytoplasm |
| CA.PGAv.1.6.scaffold26.14 | GPX7 | 28.82 | 10 | 38.82 | 22.35 | Chloroplast |
| CA.PGAv.1.6.scaffold559.5 | GPX8 | 25.52 | 9.21 | 39.33 | 25.94 | Chloroplast |
