# Supplementary material for: Characterization of GPX Gene Family in Pepper (Capsicum annuum L.) under Abiotic Stress and ABA Treatment
Source: Int J Mol Sci. 2024 Jul 30;25(15):8343. doi: 10.3390/ijms25158343 (PMC11313330; doi:10.3390/ijms25158343)
Supplement: Supplementary file 1 [file ijms-25-08343-s001.zip › Table S2.pdf]

Table S2 Ka/Ks values of *CaGPX* gene pairs in peppers and *Arabidopsis*.

Paralogous pairs	Ka	Ks	Ka/Ks	Duplication Date(MY)	Duplicate type
CaGPX1-AtGPX8	0.2384	2.8084	0.0849	230.20	Segmental
CaGPX1-AtGPX6	0.1313	2.3908	0.0549	196.00	Segmental
CaGPX3-AtGPX3	0.2516	1.6063	0.1566	131.67	Segmental
CaGPX5-AtGPX5	0.1969	1.9507	0.1010	159.89	Segmental

For each gene pair, the Ks value was translated into divergence time in millions of years based on a rate of 6.1×10-9 substitutions per site per year. The divergence time (T) was calculated as T=Ks/(2×6.1×10-9)×10-6 Mya.
