# Supplementary material for: Characterization of GPX Gene Family in Pepper (Capsicum annuum L.) under Abiotic Stress and ABA Treatment
Source: Int J Mol Sci. 2024 Jul 30;25(15):8343. doi: 10.3390/ijms25158343 (PMC11313330; doi:10.3390/ijms25158343)
Supplement: Supplementary file 1 [file ijms-25-08343-s001.zip › Table S3.pdf]

Table S3 Conserved motifs in the amino acid sequences of CaGPX proteins.

Motif	Width Multilevel	consensus sequence
1	50	EILAFPCNQFGGQEPGTNEEIKQFACTRFKAEFPIFDKVDVNGPNAAPLY
2	50	HDFTVKDIKGKDVDLSIYKGKVLLIVNVASKCGLTNSNYTELSZLYEKYK
3	50	FLKSSKGGFLGDAIKWNFTKFLVDKEGKVVERYAPTTSPLSIEKDIQKLL
4	6	MAASSS
5	11	VYARAAAEKSV
6	7	YRHPSCC
7	26	RTWAAASPFGSAAVPLVSPPSLRAPP
8	6	NSHRFJ
9	6	RRAHPL
10	14	QAELLGYHY
