# Supplementary material for: Characterization of GPX Gene Family in Pepper (Capsicum annuum L.) under Abiotic Stress and ABA Treatment
Source: Int J Mol Sci. 2024 Jul 30;25(15):8343. doi: 10.3390/ijms25158343 (PMC11313330; doi:10.3390/ijms25158343)
Supplement: Supplementary file 1 [file ijms-25-08343-s001.zip › Table S8.pdf]

Table S8 Primer sequences used for RT-PCR and vector construction.

Gene Name	Forward Primer Sequence (5'-3')	Reverse Primer Sequence(5'-3')
CaGPX1	AGGTGGGTTCTTTGGTGATGGTATC	GCTGGAGAAGTGGTTGGAGAGTAG
CaGPX2	ACTGCCCAAACCTCTCCACTTG	CGGAACCTTGCTCAGGAACATTG
CaGPX3	GTGGTTTCCTTGGAAGTGCTGTC	ACTGAAGCGGTGGTGTCTTGG
CaGPX4	TGTCGTCAATGTCGCTTCCAAATG	CCTCACTTGTCCCAGGCTCTTG
CaGPX5	GTTCAAATGCGGCACCAGTCTAC	TGAGCCATAGCGTCGGACAAC
CaGPX6	CAGCCAACCAGAGAAGCAACAATC	ACCGCATTTGGACGCAACATTG
CaGPX7	TCGTGAATGTTGCTTCCAAGTGTG	ATCATTGGTCCCTGGCTCTTCTTC
CaGPX8	ATGCTAGAGCTGCTACTGAGAAGAC	GTTGTCAAGCCACATCTGGAAGC
actin	GGAGTAATGGTAGGAATGGG	TTGCTGACAATACCATGCTC

Gene Name	Forward Primer Sequence (5'-3')	Reverse Primer Sequence(5'-3')
CaGPX1	AGGTGGGTTCTTTGGTGATGGTATC	GCTGGAGAAGTGGTTGGAGAGTAG
CaGPX4	TGTCGTCAATGTCGCTTCCAAATG	CCTCACTTGTCCCAGGCTCTTG
